# The pore-forming subunit MCU of the mitochondrial Ca^2+^ uniporter is required for normal glucose-stimulated insulin secretion in vitro and in vivo in mice

**DOI:** 10.1007/s00125-020-05148-x

**Published:** 2020-04-29

**Authors:** Eleni Georgiadou, Elizabeth Haythorne, Matthew T. Dickerson, Livia Lopez-Noriega, Timothy J. Pullen, Gabriela da Silva Xavier, Samuel P. X. Davis, Aida Martinez-Sanchez, Francesca Semplici, Rosario Rizzuto, James A. McGinty, Paul M. French, Matthew C. Cane, David A. Jacobson, Isabelle Leclerc, Guy A. Rutter

**Affiliations:** 1 Section of Cell Biology and Functional Genomics, Division of Diabetes, Endocrinology and Metabolism, Department of Metabolism, Digestion and Reproduction, Imperial College London, Du Cane Road, London, W12 0NN UK; 2 Department of Molecular Physiology and Biophysics, Vanderbilt University, Nashville, TN USA; 3 Photonics Group, Department of Physics, Imperial College London, London, UK; 4 Department of Biomedical Sciences, University of Padova, Padua, Italy

**Keywords:** Calcium, Glucose homeostasis, Insulin secretion, Mitochondria, Mitochondrial Ca^2+^ uniporter (MCU), Pancreatic beta cells, Type 2 diabetes

## Abstract

**Aims/hypothesis:**

Mitochondrial oxidative metabolism is central to glucose-stimulated insulin secretion (GSIS). Whether Ca^2+^ uptake into pancreatic beta cell mitochondria potentiates or antagonises this process is still a matter of debate. Although the mitochondrial Ca^2+^ importer (MCU) complex is thought to represent the main route for Ca^2+^ transport across the inner mitochondrial membrane, its role in beta cells has not previously been examined in vivo.

**Methods:**

Here, we inactivated the pore-forming subunit of the MCU, encoded by *Mcu*, selectively in mouse beta cells using *Ins1*^Cre^-mediated recombination. Whole or dissociated pancreatic islets were isolated and used for live beta cell fluorescence imaging of cytosolic or mitochondrial Ca^2+^ concentration and ATP production in response to increasing glucose concentrations. Electrophysiological recordings were also performed on whole islets. Serum and blood samples were collected to examine oral and i.p. glucose tolerance.

**Results:**

Glucose-stimulated mitochondrial Ca^2+^ accumulation (*p*< 0.05), ATP production (*p*< 0.05) and insulin secretion (*p*< 0.01) were strongly inhibited in beta cell-specific *Mcu*-null (β*Mcu*-KO) animals, in vitro, as compared with wild-type (WT) mice. Interestingly, cytosolic Ca^2+^ concentrations increased (*p*< 0.001), whereas mitochondrial membrane depolarisation improved in β*Mcu*-KO animals. β*Mcu*-KO mice displayed impaired in vivo insulin secretion at 5 min (*p*< 0.001) but not 15 min post-i.p. injection of glucose, whilst the opposite phenomenon was observed following an oral gavage at 5 min. Unexpectedly, glucose tolerance was improved (*p*< 0.05) in young β*Mcu*-KO (<12 weeks), but not in older animals vs WT mice.

**Conclusions/interpretation:**

MCU is crucial for mitochondrial Ca^2+^ uptake in pancreatic beta cells and is required for normal GSIS. The apparent compensatory mechanisms that maintain glucose tolerance in β*Mcu*-KO mice remain to be established.

**Electronic supplementary material:**

The online version of this article (10.1007/s00125-020-05148-x) contains peer-reviewed but unedited supplementary material, which is available to authorised users.

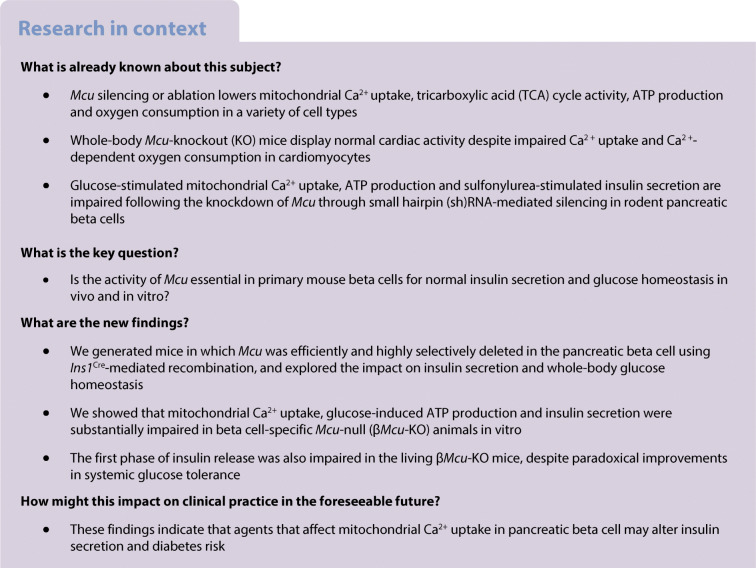



## Introduction

Defective insulin secretion underlies diabetes mellitus, a disease affecting almost one in eight of the adult population worldwide [1]. The most prevalent form of this condition is type 2 diabetes, where pancreatic beta cell failure usually, though not always, occurs in the face of insulin resistance in other tissues [2]. Current therapeutic strategies have limited efficacy and there remains a desperate need to develop new treatments to tackle this growing epidemic.

Pancreatic beta cells ensure blood glucose homeostasis by responding to a rise in circulating nutrient levels with insulin secretion. Glucose-induced increases in mitochondrial oxidative metabolism are central to the stimulation of hormone release, and drive an increase in cytosolic ATP:ADP ratio, closure of ATP-sensitive K^+^ (K_ATP_) channels, Ca^2+^ influx and exocytosis [3]. Ca^2+^ ions are also taken up by mitochondria [4, 5] and this has been suggested to activate the tricarboxylic acid (TCA) cycle and other intra-mitochondrial enzymes [6] in order to enhance the production of reducing equivalents for the electron transport chain, and ATP generation [3]. Although a number of approaches have been used previously to explore the role of intra-mitochondrial Ca^2+^ in controlling insulin secretion, the role of these ions in modifying ATP synthesis and, hence, exocytosis in these cells is still debated [7, 8].

Importantly, there is accumulating evidence to suggest that mitochondrial dysfunction in the pancreatic beta cell leads to impaired glucose-stimulated insulin secretion (GSIS) and may contribute to the development of type 2 diabetes [9]. Moreover, defective mitochondrial Ca^2+^ uptake in isolated beta cells may lead to lowered insulin secretion in response to glucolipotoxicity [10].

The mitochondrial Ca^2+^ uniporter (MCU; previously termed MCUa or CCD109A) forms the Ca^2+^-selective pore of a multi-protein MCU-complex, alongside mitochondrial calcium uptake protein (MICU)1–3, MCU regulator 1 (MCUR1) and the essential MCU regulator (EMRE [also known as single-pass membrane protein with aspartate rich tail 1 (SMDT1)], which allows Ca^2+^ entry into mitochondria [11]. In vitro and in vivo models of *Mcu* silencing or ablation have revealed a robust reduction in mitochondrial Ca^2+^ uptake associated with blunted Ca^2+^-dependent activation of the TCA cycle, oxygen consumption, ATP production [10, 12] and mitochondrial reactive oxygen species generation [13]. Whole-body *Mcu-*knockout (KO) mice display normal basal cardiac parameters even though mitochondria isolated from cardiac myocytes display impaired Ca^2 +^ uptake and Ca^2 +^-dependent oxygen consumption. Interestingly, resting ATP levels are unaltered in muscle cells in *Mcu*-KO mice, suggesting that *Mcu* depletion does not affect basal mitochondrial metabolism. *Mcu*-KO mice, similarly, display only reduced maximal muscle power in association with reduced metabolic flux and activity of the TCA cycle enzymes in skeletal muscle mitochondria [12]. Given that both cardiac and skeletal muscles are highly metabolically active tissues, it is surprising that whole-body *Mcu*-KO mice display a mild phenotype [12, 13]. However, glucose homeostasis and insulin secretion were not examined in detail in these earlier studies.

We have previously shown that reducing glucose-stimulated mitochondrial Ca^2+^ uptake in rodent pancreatic beta cells through knockdown of *Mcu* through small hairpin RNA (shRNA)-mediated silencing in vitro impairs the sustained increase in ATP:ADP ratio usually seen in response to high glucose and ablates sulfonylurea-stimulated insulin secretion [10, 14]. Similar findings were also observed in clonal beta cells [15]. However, these earlier studies provided no insight into the impact of reducing mitochondrial Ca^2+^ uptake on GSIS in vivo, nor how this may, in turn, impact the physiology of the living animal.

Tissue-specific manipulation of MCU activity provides an alternative and powerful means to examine the role of mitochondrial Ca^2+^ in a particular tissue or cell type. In the present study, we have therefore generated mice in which *Mcu* is deleted highly selectively in the pancreatic beta cell, and explored the impact of this on insulin secretion and whole-body glucose homeostasis.

## Methods

### Generation of beta cell-selective Mcu-KO mice

Two to five C57BL/6 J mice were housed per individually ventilated cage in a pathogen-free facility with a 12 h light-dark cycle and were fed ad libitum with a standard mouse chow diet (Research Diets, New Brunswick, NJ, USA) unless otherwise stated. For high-fat high-sucrose diet (HFHS) treatment (58% [wt/wt] fat and sucrose content; D12331; Research Diets), mice were placed on the diet at 5–6 weeks of age for 4 weeks prior to analysis. All in vivo procedures were approved by the UK Home Office, according to the Animals (Scientific Procedures) Act 1986.

In brief, C57BL/6 J mice bearing *Mcu* alleles with floxP sites flanking exons 11 and 12 were generated by GenOway (strain B6-*Mcu*^tm1Geno^; Grenoble, France) and bred to animals carrying Cre recombinase inserted at the *Ins1* locus (*Ins1*^Cre^), allowing efficient and beta cell-selective deletion of both *Mcu* splice variants, without recombination in the brain or confounding expression of human growth hormone. Mice bearing floxed *Mcu* alleles but lacking Cre recombinase were used as wild-type (WT) littermate controls. Possession of *Ins1*Cre alleles alone exerted no effect on glycaemic phenotype (data not shown).

### SDS-PAGE and western blotting

MCU expression was assessed in islets isolated from 8–10-week-old WT and beta cell-specific *Mcu*-null (β*Mcu*-KO) male animals. For full details, see electronic supplementary material (ESM) Methods.

### mRNA extraction and quantitative reverse transcription PCR

For measurements of mRNA levels, pancreatic islets from 8–10-week-old WT and β*Mcu*-KO males were isolated by collagenase digestion. Alongside mouse islets, RNA was extracted from liver, heart and adipose tissues. Gene expression determined by quantitative reverse transcription PCR (qRT-PCR) and normalised to *β-actin* (see ESM Methods for details; primer details are given in ESM Tables 1 and 2).

### Single-cell fluorescence imaging

Pancreatic islets were isolated from 8–10-week-old male mice, dissociated into single beta cells and plated onto glass coverslips [10, 16]. Mitochondrial Ca^2+^ uptake was measured via adenovirus-mediated delivery of a mitochondrially-targeted recombinant Ca^2+^ probe, R-GECO (multiplicity of infection [MOI]: 100; sequence obtained from Addgene [Watertown, NY, USA]; adenovirus generated using the PAdEasy system [Addgene]) [17, 18]. Cells were infected and incubated for 48 h prior to imaging in Krebs-Ringer bicarbonate buffer (140 mmol/l NaCl, 3.6 mmol/l KCl, 0.5 mmol/l NaH_2_PO_4_, 24 mmol/l NaHCO_3_ [saturated with CO_2_], 1.5 mmol/l CaCl_2_, 0.5 mmol/l MgSO_4_, 10 mmol/l HEPES and 3 mmol/l d-glucose; pH 7.4). To examine ATP:ADP changes in response to a rise in extracellular glucose concentration, dissociated beta cells were infected with an adenovirus bearing cDNA encoding the ATP sensor pGW1CMV-Perceval (MOI: 100; kindly provided by G. Yellen [Yale University, New Haven, CT, USA]) [10] and incubated for 48 h prior to fluorescence imaging. In all experiments, cells were equilibrated for at least 30 min in Krebs-Ringer bicarbonate buffer containing 3 mmol/l glucose prior to the start of acquisitions. Excitation/emission wavelengths (nm) were: 490/630 and 410/630 (Fura-Red), 530/590 (R-GECO) and 470/535 (Perceval). All imaging experiments were performed using an Olympus IX71 microscope (Olympus, Southend-on-Sea, UK) with a ×40 magnification objective, a Zyla sCMOS camera (Andor, Oxford, UK) and a polychrome IV (BioProcess, Horsham, PA, USA) excitation system with image capture at 0.2 Hz (excitation time, 50 ms).

For experiments with tetramethylrhodamine ethyl ester (TMRE), beta cells were loaded with 10 nmol/l TMRE in Krebs-Ringer bicarbonate buffer with 3 mmol/l glucose (pH 7.4) for 45 min, and re-equilibrated with 2 nmol/l TMRE for 10 min before recordings. TMRE (2 nmol/l) was present throughout and cells were excited at 550 nm. Carbonyl cyanide-4-phenylhydrazone (FCCP; 1 μmol/l) was administrated, as indicated, and imaging was performed using a Zeiss AxioObserver microscope (Zeiss, Cambridge, UK) using a× 40 1.4 numerical aperture (NA) oil objective, a Hamamatsu Flash 4 camera (Welwyn Garden City, UK) and a Colibri.2 light emitting diode (LED) excitation system (Zeiss; excitation filter 534/20 nm; emission filter 572/28 nm) at 0.3 Hz (250 ms exposure). Traces represent mean normalised fluorescence intensity over time (F/F_min_), where F_min_ is the mean fluorescence recorded at the end of the application of FCCP. Delta psi (Δ_psi_) is the difference in mean fluorescence intensity measured at the peak following administration of 17 mmol/l glucose (711–732 s) vs the minimum prior to glucose elevation (189–210 s).

### Whole-islet fluorescence imaging

Ca^2+^ imaging of whole islets was performed after loading with R-GECO (48 h post-isolation) in Krebs-Ringer bicarbonate buffer or cytosolic Cal-520 acetoxymethyl (AM; 2 μmol/l; 24 h post-isolation; Stratech, Cambridge, UK) in modified Krebs-Ringer bicarbonate buffer (140 mmol/l NaCl, 3.6 mmol/l KCl, 0.5 mmol/l NaH_2_PO_4_, 2 mmol/l NaHCO_3_ [saturated with CO_2_], 1.5 mmol/l CaCl_2_, 0.5 mmol/l MgSO_4_, 10 mmol/l HEPES; pH 7.4) containing 3 mmol/l or 17 mmol/l glucose, 17 mmol/l glucose with 0.1 mmol/l diazoxide (Sigma-Aldrich, Dorset, UK), or 20 mmol/l KCl. Images were captured at 0.5 Hz on a Zeiss Axiovert microscope equipped with a ×10 0.3–0.5 NA objective, a Hamamatsu image-EM camera coupled to a Nipkow spinning-disk head (Yokogawa CSU-10; Runcorn, UK) and illuminated at 490 nm or 530 nm. Data were analysed using ImageJ (https://imagej.nih.gov/ij/download.html, accessed 15 February 2020) with a purpose-designed macro (available upon request).

### IPGTT and IPITT tests and measurement of insulin secretion in vivo

To investigate glucose or insulin tolerance, male or female mice (aged 8–24 weeks) were fasted and injected with glucose or insulin i.p. Glucose was measured in tail vein blood using a glucometer. For in vivo insulin secretion experiment, fasted male mice were administered glucose either i.p. or by oral gavage; plasma insulin was measured using an ELISA kit. See ESM Methods for details.

### In vitro insulin secretion

Insulin secretion assays were performed in triplicate on size-matched islets, isolated from male mice (8–10 weeks of age) and incubated for 1 h in modified Krebs-Ringer bicarbonate buffer containing 3 mmol/l glucose. Subsequently, islets were either perifused (~50 islets/chamber) or batch incubated (10 islets/well) for 30 min in Krebs-Ringer solution with either 3 mmol/l or 17 mmol/l glucose, 10 mmol/l glucose supplemented with 100 nmol/l exendin-4 (Wuxi Apptec, Shanghai, China), or the glucose-dependent insulinotropic peptide (GIP; Wuxi Apptec). Secreted and total insulin content were quantified using a homogeneous time-resolved fluorescence (HTRF) insulin kit (Cisbio, Codolet, France) in a PHERAstar reader (BMG Labtech, Aylesbury, UK), following the manufacturer’s guidelines. Data are presented as secreted insulin/insulin content.

### Electrophysiology

Voltage-dependent calcium channel (VDCC) currents were recorded from dispersed mouse beta cells, as previously described [19]. See ESM Methods for details.

### Beta cell mass

Whole pancreatic optical projection tomography (OPT), to 19 μm resolution, was performed, as described [20].

### Statistical analysis

Data are expressed as mean ± SEM. Significance was tested by Student’s two-tailed *t* test or one- or two-way ANOVA with Sidak’s or Bonferroni multiple comparison test for comparisons of more than two groups, using GraphPad Prism 8 software (San Diego, CA, USA). *p*< 0.05 was considered significant. Experiments were not randomised or blinded.

## Results

### *Mcu* ablation from pancreatic beta cells attenuates GSIS in vitro

**Fig. 1:**
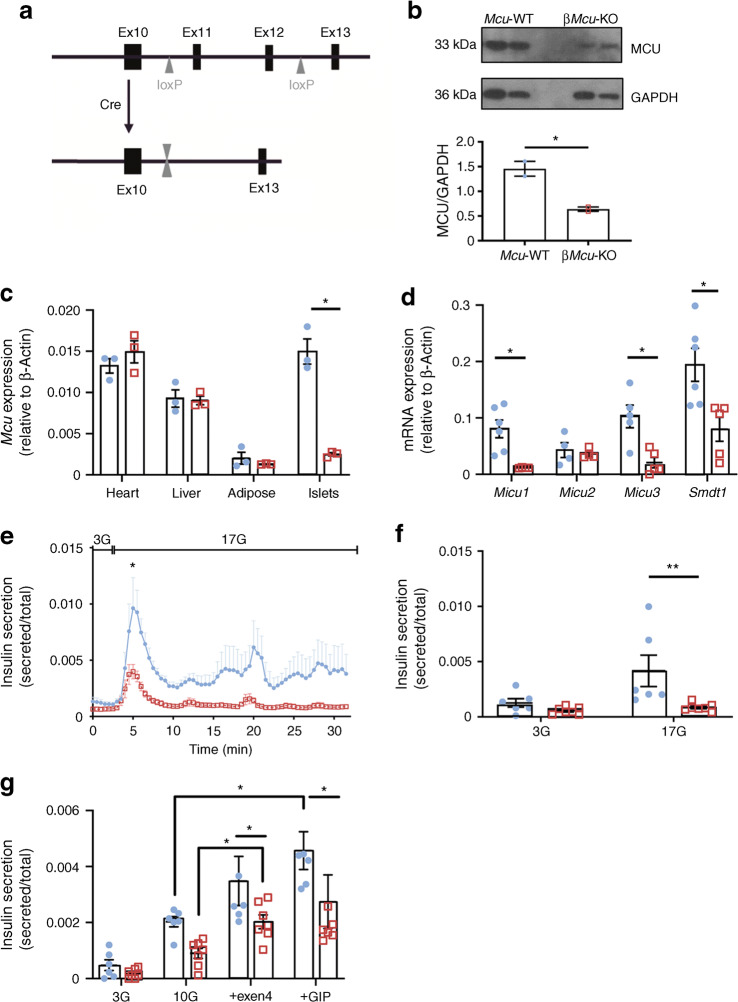
Isolated islets from male β*Mcu*-KO mice display attenuated GSIS in vitro. (**a**) Gene deletion was achieved by breeding mice carrying *Mcu* alleles with floxP (loxP) sites flanking exon (Ex) 11 and Ex12 with mice bearing Cre recombinase inserted at the *Ins1* locus. (**b**) Western blot analysis demonstrating efficient MCU deletion (*n =* 2 mice per genotype in three independent experiments) in isolated islets. (**c**) qRT-PCR quantification of *Mcu* expression in heart, liver, adipose tissue and islets relative to β-actin (*n =* 3 mice per genotype in two independent experiments). (**d**) qRT-PCR quantification of *Micu1–3* and *Smdt1* expression in islets relative to β-actin (*n =* 4–6 mice per genotype in two independent experiments). (**e**) Insulin secretion from islets isolated from male β*Mcu*-WT and KO mice during perifusion and (**f**) serial incubations of islets in batches, at 3 mmol/l glucose (3G) or 17 mmol/l glucose (17G). In (**e**), a significant decrease in insulin secretion was observed in islets isolated from KO mice during the first peak (4–8 min) vs WT mice (*n =* 4–5 mice per genotype in three independent experiments). In (**f**), a significant decrease was observed between genotypes for insulin secretion stimulated with 17G, as compared with WT mice (*n =* 6–7 mice per genotype in three independent experiments). (**g**) Insulin secretion from islets during serial incubations in batches, at 3G, 10 mmol/l glucose (10G), or 100 nmol/l exendin-4 (exen4) or GIP in presence of 10G (*n =* 5–7 mice per genotype in three independent experiments). Blue, WT mice; red, β*Mcu*-KO mice. Data are presented as mean ± SEM.*p< 0.05; **p< 0.01, analysed by unpaired two-tailed Student’s *t* test in (**a**, **e**, **f**) or by two-way ANOVA test and Sidak’s multiple comparisons test in (**c**, **d**, **g**)

Mice bearing *Mcu* alleles with floxP sites flanking exons 11 and 12 were generated and bred to animals carrying Cre recombinase inserted at the *Ins1* locus (*Ins1*^Cre^) (Fig. 1a) [21]. MCU deletion in islets was confirmed by western blotting (Fig. 1b) and qRT-PCR in islets, heart, liver and adipose tissues (Fig. 1c). MCU immunoreactivity was decreased by 50–60% in islets (*p*< 0.05; Fig. 1b), suggesting that Ca^2+^ influx was impaired but not completely abolished in β*Mcu*-KO mice. Relative to *β-actin*, expression of the *Mcu* transcript in islets from KO mice was decreased by ~80% vs control islets (*p*< 0.05; Fig. 1c), whereas no significant differences were identified in other mouse tissues. This level of reduction is consistent with near-complete elimination of *Mcu* mRNA from beta cells, assuming a beta:alpha cell ratio of ~3:1 [22] and similar levels of *Mcu* expression in each cell type in islets from WT animals [23].

Next, to determine whether the genes encoding other pore-forming or regulatory proteins (*Micu1–3* and *Smdt1*) of the uniporter were disrupted following *Mcu* deletion in the islets, their mRNA expression levels were quantified (Fig. 1d). Interestingly, *Micu1*, *Micu3* and *Smdt1* mRNA levels also significantly decreased (*p*< 0.05) in islets of KO mice.

We next explored the consequences of *Mcu* KO with regards to GSIS from isolated islets from β*Mcu*-KO mice. In perifusion experiments, islets from β*Mcu*-KO mice displayed a significant blunting in the secretory response to elevated glucose, with the attenuation in insulin release most evident at high glucose concentrations (17 mmol/l), as determined at the first peak of insulin release (*p*< 0.05; Fig. 1e). These results were confirmed by independent experiments using batch incubation of islets (*p*< 0.01; Fig. 1f). In contrast, islets from β*Mcu*-KO mice displayed no difference vs control islets in insulin secretion stimulated by depolarisation with 20 mmol/l KCl in both perifusion or batch incubation systems (data not shown). Finally, to examine whether the decline in GSIS in islets from β*Mcu*-KO was linked with an impaired incretin response, GIP or exendin-4 were used to co-stimulate insulin secretion at 10 mmol/l glucose. (Fig. 1g). Despite enhanced insulin secretion in both groups (in comparison with 10 mmol/l glucose alone; *p*< 0.05), islets from β*Mcu*-KO mice showed impaired GSIS (*p*< 0.05).

### *Mcu* deletion from pancreatic beta cells impairs glucose-stimulated mitochondrial but not cytosolic Ca^2+^ uptake

**Fig. 2:**
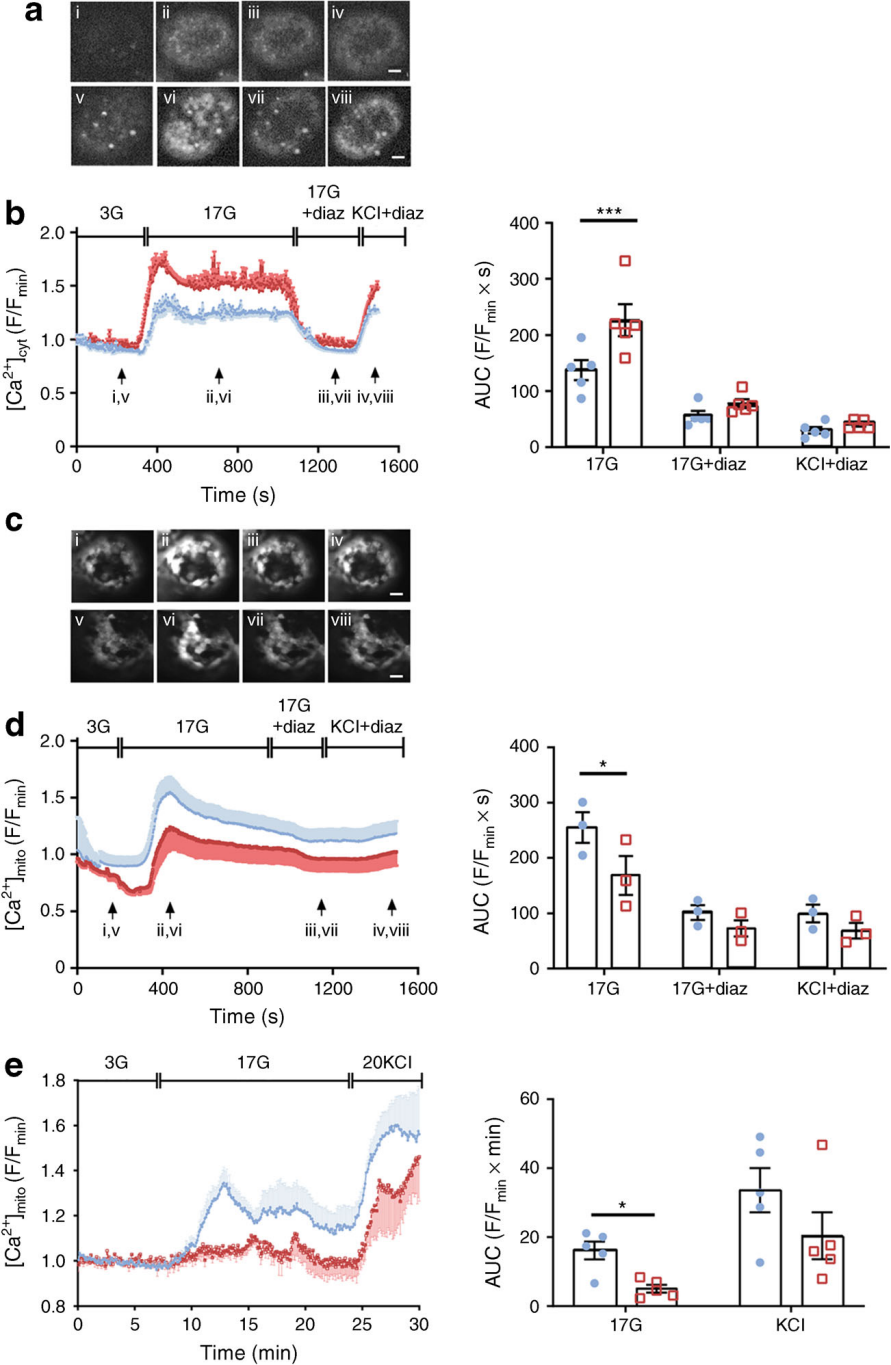
*Mcu* deletion from pancreatic beta cells diminishes in vitro mitochondrial Ca^2+^ uptake in dissociated islets but not [Ca^2+^]_cyt_ in whole islets. (**a**) Each snapshot of isolated WT (i–iv) and KO-derived (v–viii) islets was taken during the time points indicated by the respective arrow in (**b**). Scale bar, 50 μm. See also ESM Video 1. (**b**) [Ca^2+^]_cyt_ changes in response to 17 mmol/l glucose (17G; with or without diazoxide [diaz]) and 20 mmol/l KCl + diaz were assessed following Cal-520 uptake in whole islets. Traces represent mean normalised fluorescence intensity over time (F/F_min_). 3G, 3 mmol/l glucose. The corresponding AUC is also presented (*n =* 5 individual trials in two independent experiments with *n* = 3 mice per genotype; 17G AUC measured between 300 s and 1120 s, 17G + diaz AUC measured between 1121 s and 1385 s), and KCl + diaz AUC measured between 1386 s and 1500 s). (**c**) Each snapshot of isolated WT (i–iv) and KO-derived (v–viii) islets was taken during the time points indicated by the respective arrow in (**d**). Scale bar, 50 μm. See also ESM Video 2. (**d**) [Ca^2+^]_mito_ changes in response to 17G (with or without diaz) and 20 mmol/l KCl + diaz were assessed in islets following R-GECO infection. Traces represent mean normalised fluorescence intensity over time (F/F_min_). The corresponding AUC is also shown (*n =* 6 individual trials in two independent experiments with *n* = 3 mice per genotype; 17G AUC measured between 300 s and 900 s, 17G + diaz AUC measured between 901 s and 1200 s, and KCl + diaz AUC measured between 1201 s and1500 s). (**e**) [Ca^2+^]_mito_ dynamics in response to 17G and 20 mmol/l KCl were assessed in dissociated beta cells following R-GECO infection. The corresponding AUC is also shown (*n =* 5 individual trials in two independent experiments with *n* = 3 mice per genotype; 17G AUC measured between 8 min and 15 min). Blue, WT mice; red, β*Mcu*-KO mice. Whole or dissociated islets were isolated from 8–10-week-old male mice maintained on standard chow diet. Data are presented as mean ± SEM. *p< 0.05; ***p< 0.001, assessed by two-way ANOVA and Sidak’s correction for multiple comparisons

To determine how beta cell-specific *Mcu* deletion in mitochondria might affect cytosolic Ca^2+^ dynamics in whole islets, the latter were explored using the Ca^2+^-sensitive dye Cal-520 by live-cell fluorescence microscopy. After pre-incubation in the presence of low (3 mmol/l) intracellular glucose, increases in cytosolic Ca^2+^ concentration ([Ca^2+^]_cyt_) were provoked in islets by stimulation with high (17 mmol/l) glucose. A depolarising K^+^ concentration (20 mmol/l KCl) and the K_ATP_ channel opener diazoxide were then deployed together to bypass glucose regulation of the latter (Fig. 2a,b). Interestingly, [Ca^2+^]_cyt_ in whole islets from β*Mcu*-KO mice was significantly increased in response to glucose, in comparison with WT animals (AUC, *p*< 0.001; Fig. 2b).

Since MCU provides the main route for Ca^2+^ entry into mitochondria in other cell types [24], we then determined the impact of deleting *Mcu* on this process. Changes in mitochondrial free Ca^2+^ concentration ([Ca^2+^]_mito_) were investigated in whole or dissociated islets infected with a genetically encoded mitochondrial Ca^2+^ indicator (R-GECO) [18]. *Mcu* deletion attenuated glucose-stimulated increases in [Ca^2+^]_mito_ in intact whole islets, where a significant difference in response to high glucose was observed between the two groups (AUC, *p*< 0.05; Fig. 2c,d). [Ca^2+^]_mito_ was also assessed in dissociated islets and by determination of the mean AUC at 17 mmol/l glucose (AUC, *p*< 0.05; Fig. 2e). Similar to the results obtained in whole islets, [Ca^2+^]_mito_ accumulation in response to high glucose was significantly reduced in the beta cells from KO mice.

### *Mcu* deletion from pancreatic beta cells reduces mitochondrial ATP production, whereas mitochondrial membrane depolarisation decreases in response to glucose

Given the significant reduction in GSIS observed in islets from β*Mcu*-KO mice in vitro, despite improved cytosolic Ca^2+^ dynamics, we next sought to determine whether an alteration in glucose metabolism might contribute to the attenuated insulin secretion observed. Since both fluorescent approaches to measuring [Ca^2+^]_mito_ performed on dissociated and whole islets exhibited a comparable response to glucose, the following experiments were completed on dissociated islets. This allowed us to investigate changes in ATP production, and plasma membrane potential or mitochondrial membrane potential (Δψ_m_), in response to high glucose at the beta cell level.

**Fig. 3:**
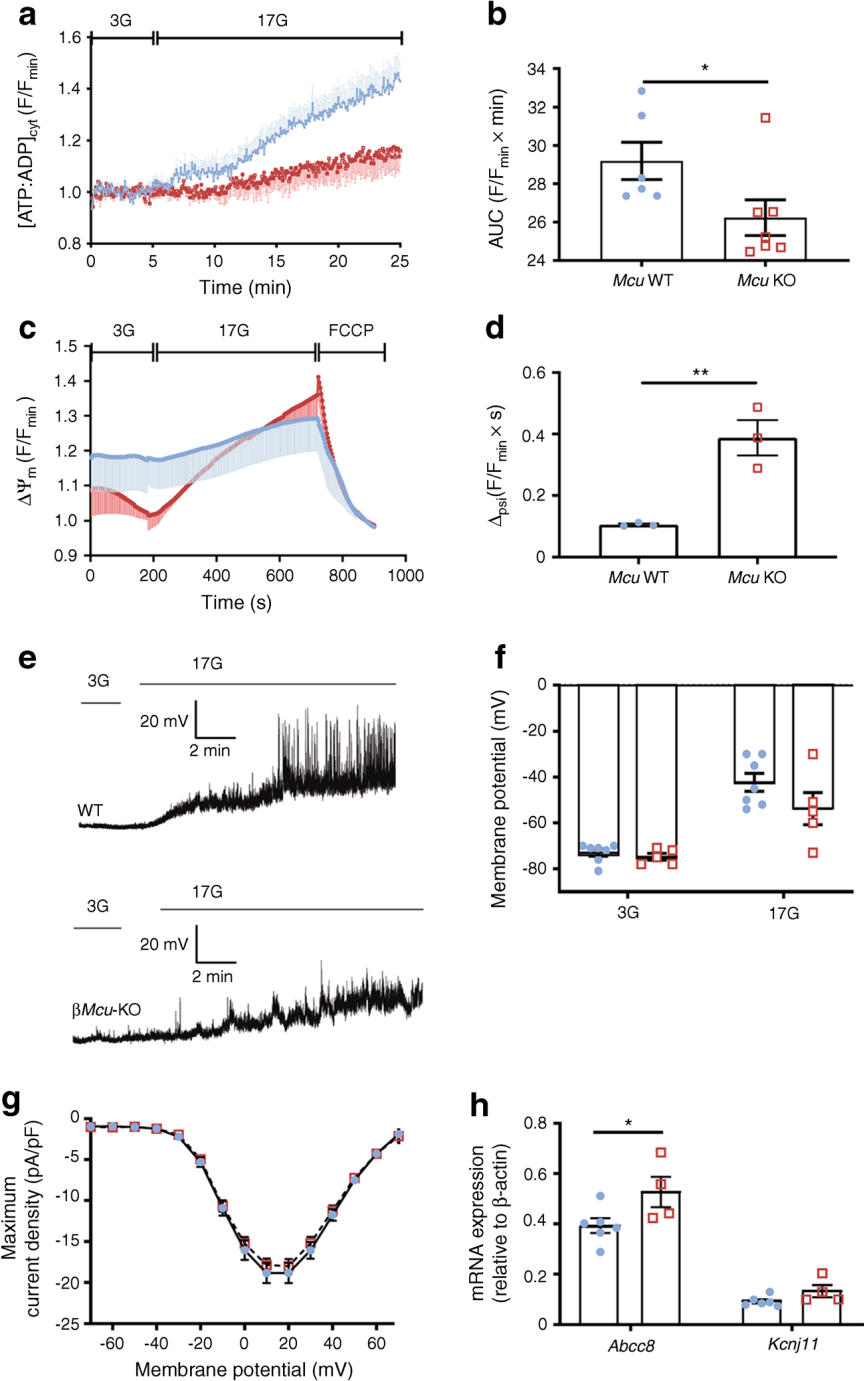
*Mcu* ablation from pancreatic beta cells diminishes ATP production and mitochondrial membrane depolarisation in response to high glucose. (**a**) Changes in the cytoplasmic ATP:ADP ratio ([ATP:ADP]_cyt_) in response to 17 mmol/l glucose (17G) was examined in dissociated beta cells using the ATP sensor Perceval. (**b**) AUC values corresponding to (**a**) (*n =* 6–7 individual trials in two independent experiments, 3 mice per genotype; unpaired two-tailed Student’s *t* test). (**c**) Cells were loaded with TMRE to measure changes in Δψ_m_, and perifused with 3 mmol/l glucose (3G), 17G or FCCP as indicated. Traces represent normalised fluorescence intensity over time (F/F_min_). (**d**) Δ_psi_ between 189–210 s (under 3G exposure) and 711–732 s (under 17G exposure) was determined from the data shown in (**c**) and presented as mean ± SEM (data points from *n =* 3 mice per genotype from two independent experiments; unpaired two-tailed Student’s *t* test). (**e**) Representative current-clamp recordings of individual beta cells from WT and β*Mcu*-KO mice, displaying the plasma membrane potential response from 3G to 17G. (**f**) Mean plasma membrane potential responses (*n =* 5–7 trials, 3 mice per genotype; two-way ANOVA test). (**g**) Activation of beta cell VDCCs in response to 17G and indicated voltage steps (*n =* 23–24 islets, *n* = 3 mice per genotype of two independent experiments; two-way ANOVA test). (**h**) qRT-PCR quantification of *Kcnj11* and *Abcc8* expression (*n =* 4–6 mice per genotype in two independent experiments; unpaired two-tailed Student’s *t* test and Mann–Whitney correction). Blue, WT mice; red, β*Mcu*-KO mice. Islets were isolated from 8–10-week-old male mice maintained on standard chow diet. Data are presented as mean ± SEM. *p< 0.05; **p< 0.01

Real-time fluorescence imaging of the ATP sensor, Perceval [10], showed a rise in the ATP:ADP ratio in control beta cells by a step increase in glucose from 3 mmol/l to 17 mmol/l [10]. This change was significantly blunted in β*Mcu*-KO beta cells (AUC, *p*< 0.05; Fig. 3a,b). This was accompanied by a potentiation in Δψ_m_ (polarised state) in response to high glucose, as assessed by monitoring TMRE fluorescence Δ_psi_ in β*Mcu*-KO and control mouse beta cells (*p*< 0.01; Fig. 3c,d).

Altered ATP production in response to high glucose is expected to affect the activity of K_ATP_ channels [25]. Assessed in single beta cells using perforated patch-clamp electrophysiology [10], the extent of plasma membrane-potential depolarisation in response to a step increase in extracellular glucose from 3 mmol/l to 17 mmol/l did not differ significantly between β*Mcu*-KO and control beta cells, although there was a weaker depolarisation in KO cells (Fig. 3e,f). VDCC currents, measured by whole-cell voltage clamp [19], displayed no apparent differences in response to 17 mmol/l glucose (Fig. 3g). Interestingly, expression of the K_ATP_ channel subunit *Abcc8* was significantly elevated in islets from β*Mcu*-KO mice (AUC, *p*< 0.05; Fig. 3h) and may thus, contribute to the albeit non-significant, reductions in electrical activity observed (Fig. 3e,f).

### Lowered beta cell mass in *Mcu*-KO mice

**Fig. 4:**
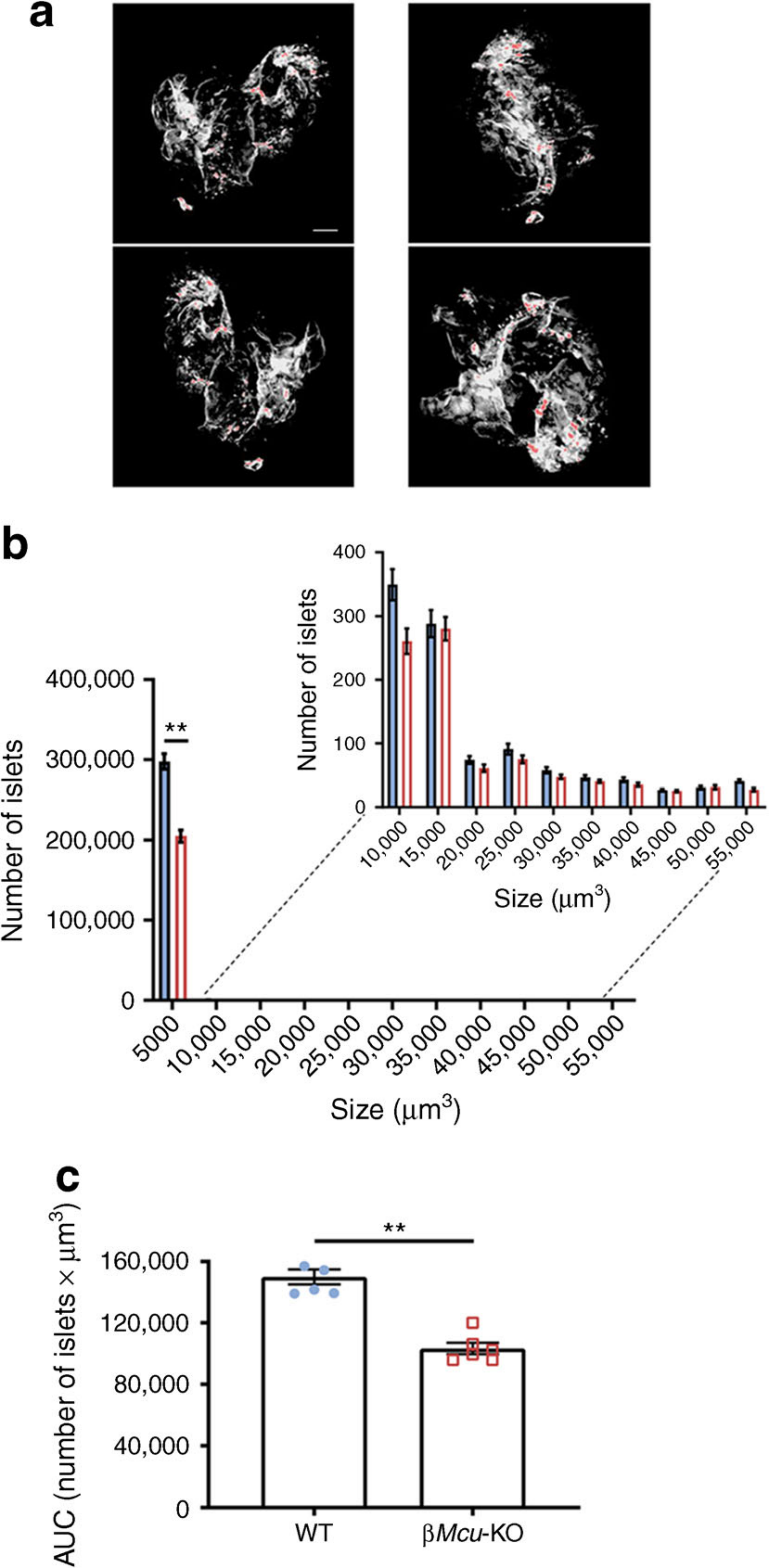
Effect of *Mcu* deletion on beta cell mass. (**a**) OPT images of representative pancreases stained with insulin (pseudo-colour, red) to indicate islets of different sizes. Scale bar, 500 μm. (**b**) Quantification of the number of islets indicates a significant decrease in smaller islets in male β*Mcu*-KO mice (*n =* 6 animals per genotype in three independent experiments). (**c**) Changes in the overall number of islets (*n* = 5–6 animals per genotype in three independent experiments). Blue, WT mice; red, β*Mcu*-KO mice. Islets were isolated from 8–10-week-old male mice maintained on standard chow diet. Data are presented as mean ± SEM. **p< 0.01, analysed by unpaired two-tailed Student’s *t* test and Mann–Whitney correction

Analysis using OPT (Fig. 4a) revealed that pancreases from β*Mcu*-KO mice displayed decreased numbers of islets at the lowest extreme of the size spectrum (*p*< 0.01), in comparison with WT mice (Fig. 4b) and a decrease in total beta cell mass (AUC, *p*< 0.01; Fig. 4c).

### Loss of *Mcu* from pancreatic beta cells does not alter body mass or fed blood glucose levels but impairs GSIS in vivo

We next explored the role of beta cell MCU in the control of insulin secretion and in vivo glucose homeostasis. Male β*Mcu*-KO animals displayed normal growth and weight changes from 6 to 24 weeks of age (ESM Fig. 1a). However, a slight, but significant, increase (*p*< 0.05) in weight gain was observed from 20 to 24 weeks of age in βMcu-KO compared with control mice. We observed no differences in random fed blood glucose levels between male β*Mcu*-KO and control animals at all ages examined (ESM Fig. 1b). No genotype-dependent differences in the above metabolic parameters were observed in female mice at any age (ESM Fig. 1c, d; ESM Fig. 2a-d).

**Fig. 5:**
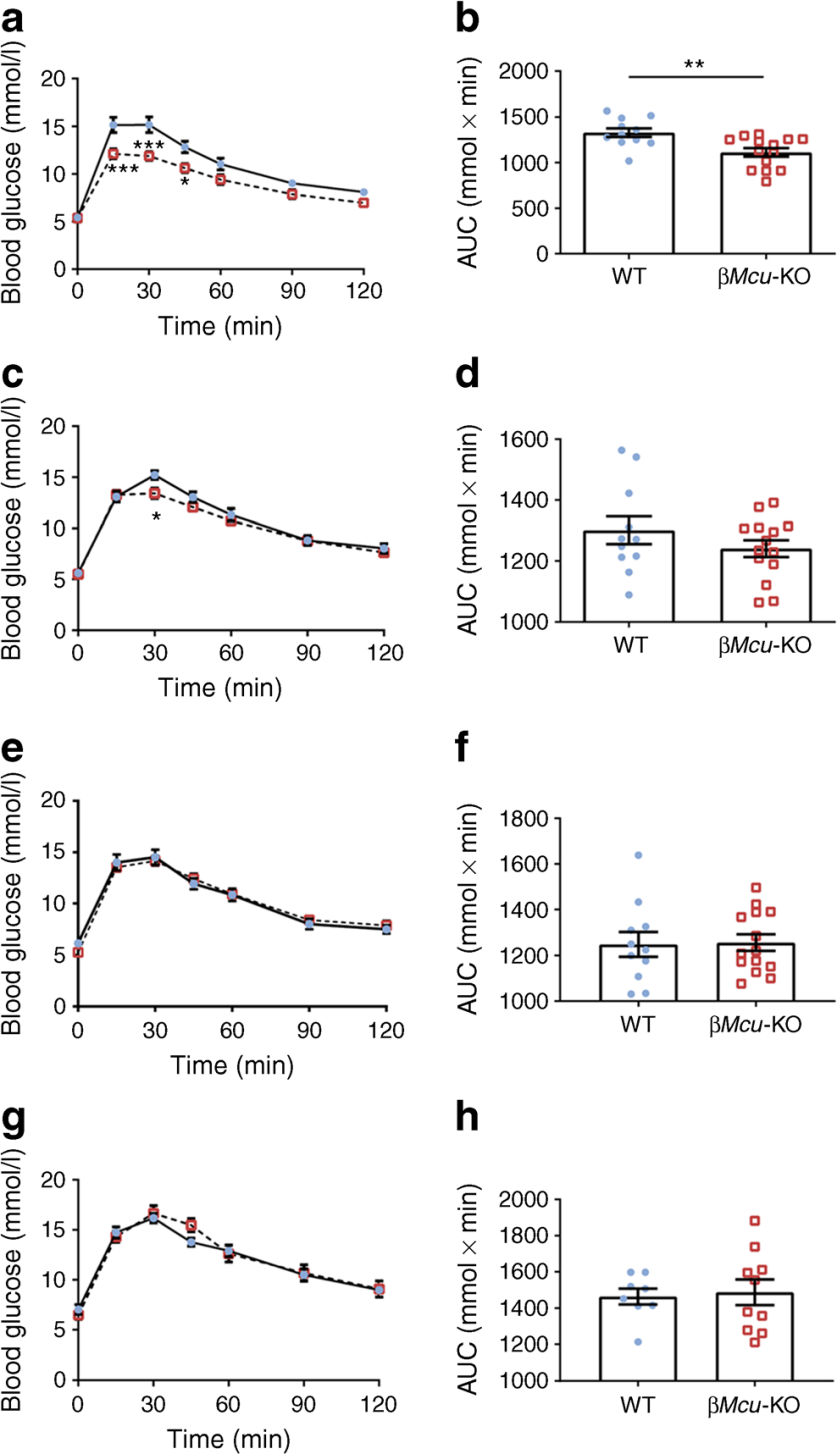
Male β*Mcu*-KO mice display slightly improved glucose tolerance. Glucose tolerance was measured in β*Mcu*-KO mice and littermate controls (WT) by IPGTT (1 g/kg body weight) at (**a**,**b**) 8, (**c**,**d**) 12, (**e**,**f**) 16 and (**g**,**h**) 24 weeks of age. The corresponding AUC is shown for each graph (*n =* 8–14 mice per genotype in four or five independent experiments). Blue, WT mice; red, β*Mcu*-KO mice. Islets were isolated from 8–10-week-old male mice maintained on standard chow diet. Data are presented as mean ± SEM. *p< 0.05; **p< 0.01; ***p< 0.001 as indicated or WT vs KO mice at the time points indicated, analysed by two-way ANOVA test

Glucose tolerance was investigated in β*Mcu*-KO and WT mice by i.p. injection of 1 g/kg body weight glucose (IPGTT) at 8, 12, 16 and 24 weeks of age. A small but significant improvement in glucose tolerance was observed in male β*Mcu*-KO mice vs controls at 8 (*p*< 0.001) and 12 (*p*< 0.05) weeks of age (Fig. 5a–d). Older male β*Mcu*-KO animals displayed unaltered glucose tolerance (Fig. 5e–h) vs WT mice.

**Fig. 6:**
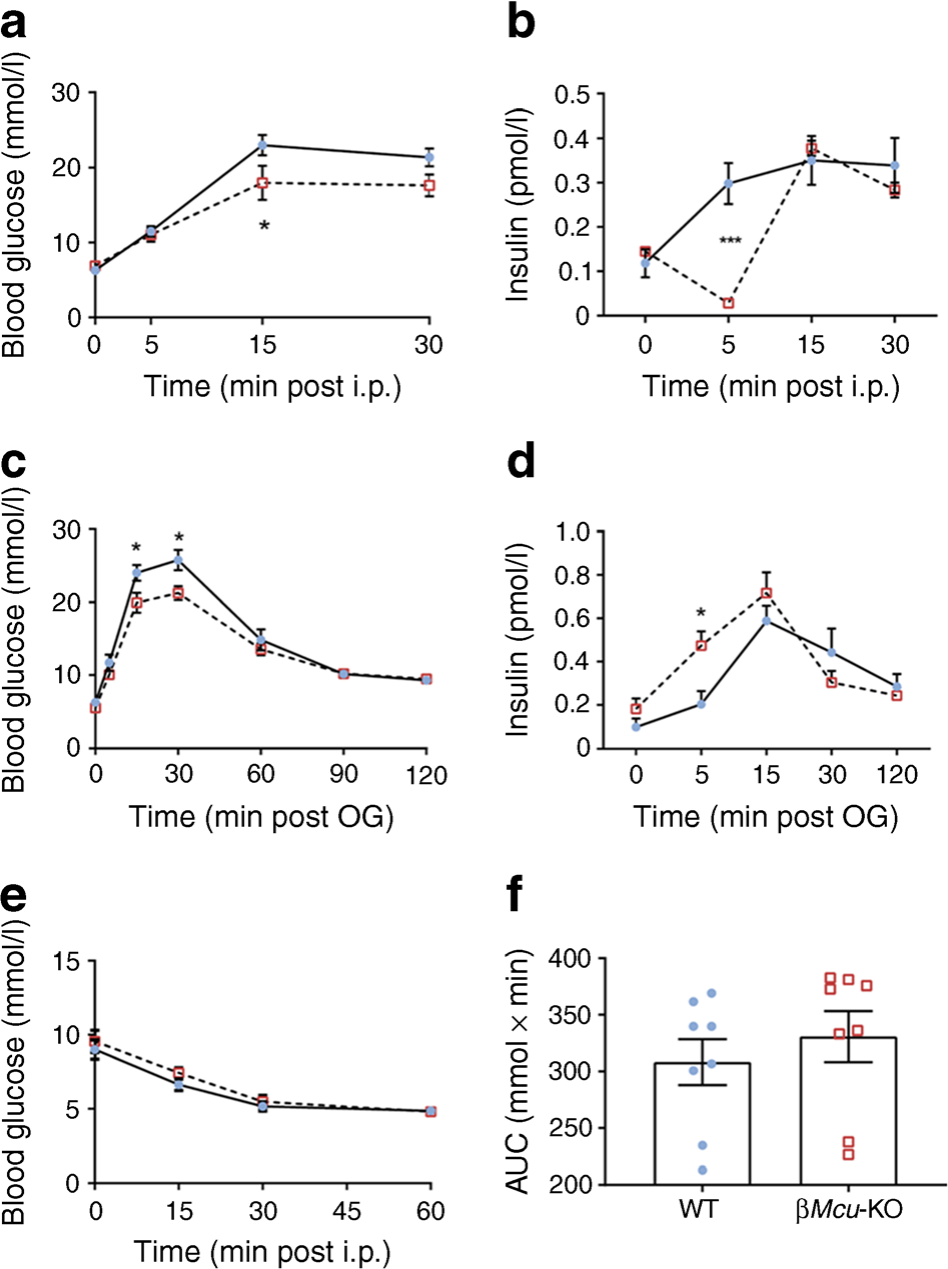
β*Mcu*-KO mice display enhanced glucose tolerance following i.p glucose administration or glucose administration by oral gavage (OG). (**a**) Blood glucose and (**b**) glucose-induced insulin secretion (using 3 g/kg body weight) were assessed in 8–10-week-old male β*Mcu*-KO and WT mice (*n =* 6–9 mice per genotype in three or four independent experiments; two-way ANOVA test). (**c**) Plasma glucose and (**d**) insulin, during OGTT in male β*Mcu*-KO and WT mice (*n =* 7–9 mice per genotype in two or four independent experiments; two-way ANOVA test). (**e**) Challenging 8–10-week-old male β*Mcu*-KO mice with a 0.75 U/kg body weight insulin injection resulted in normal insulin sensitivity as compared with WT mice. (**f**) Corresponding AUC for (**e**) is also shown (*n =* 8 mice per genotype in three independent experiments; unpaired two-tailed Student’s *t* test and Mann–Whitney correction). All mice were maintained on a standard chow diet. Blue, WT mice; red, β*Mcu*-KO mice. Data are presented as mean ± SEM. *p< 0.05; ***p< 0.001 for WT vs KO mice at the time points indicated

To assess GSIS in vivo, 8–10-week-old male mice were challenged with 3 g/kg body weight glucose and plasma insulin was sampled at 0, 5, 15 and 30 min. Although improved glucose tolerance was observed in β*Mcu*-KO animals post-15 min i.p. glucose administration (Fig. 6a), a dramatic reduction (*p*< 0.001) in insulin release was observed 5 min post-glucose injection (Fig. 6b). β*Mcu*-KO animals also displayed improved oral glucose tolerance at 15 and 30 min post-oral gavage (*p*< 0.05; Fig. 6c) and increased insulin secretion at 5 min (*p*< 0.05; Fig. 6d) vs WT littermates. No differences in i.p. insulin tolerance (Fig. 6e,f) or C-peptide levels (not shown) were observed between WT and KO mice.

Finally, to impose a metabolic stress, β*Mcu*-KO mice and control littermates were maintained on a HFHS diet and subjected to IPGTTs and IPITTs, as above. No differences in blood glucose levels or insulin secretion were observed between phenotypes (ESM Fig. 3a, b). In addition, no genotype-dependent differences in C-peptide secretion were apparent (not shown).

## Discussion

Using a novel mouse model to achieve highly efficient and selective ablation of *Mcu* in beta cells, we show that mitochondrial Ca^2+^ uptake can play an important role in the first phase of insulin secretion in the living mouse.

Mitochondria are highly dynamic organelles, critical for maintaining normal beta cell function and secretory responses to glucose [26–28]. As mitochondrial dynamics and biogenesis are impaired in these cells in the face of insulin resistance and in type 2 diabetes [9], it is conceivable that preserving the normal function of mitochondria may slow the loss of normal insulin secretion and disease progression [29, 30].

Recent findings have demonstrated the importance of MCU for Ca^2+^ uptake into mitochondria in several cell types and have established this as the most important route for Ca^2+^ accumulation into these organelles [11]. Analysis of published RNA sequencing (RNASeq) data [31] revealed that both long (containing exon 6) and short protein-coding *Mcu* splice variants are present in mouse beta cells. To ensure efficient deletion of each splice variant, we targeted exons 11 and 12 and neighbouring intronic sequences, ensuring the removal of the C-terminal coiled coil and trans-membrane domains of the protein. As a result, near-complete elimination of the mRNA encoding *Mcu* (likely reflecting nonsense-mediated decay), but also of other functional subunits of the *Mcu* pore, was accomplished throughout the beta cell population, without affecting other tissues, such as the heart, liver or adipose tissues. Reduction in the mRNA expression of the pore gatekeeping proteins *Micu1*, *Micu3* [32] and *Smdt1,* responsible for maintaining the pore architecture [33], indicate that deletion of *Mcu* can be linked to an impaired Ca^2+^ flux within the mitochondrial matrix.

In agreement with previous studies using RNA silencing [10, 15], *Mcu* deletion at the genomic level attenuated GSIS and mitochondrial Ca^2+^ uptake in response to high glucose in dissociated and whole islets [10]. Of note, β*Mcu*-KO-derived whole islets labelled with R-GECO showed a reduced mitochondrial Ca^2+^ uptake, demonstrating that the main pathway of Ca^2+^ entry into the organelles is significantly impaired in KO mice during the first phase of glucose challenge. On the other hand, the response to depolarisation by KCl was less markedly affected, possibly reflecting the opening of other mitochondrial Ca^2+^ transporters/channels at high cytosolic [Ca^2+^] [34]. These may include ryanodine receptors [35] or the rapid mode of mitochondrial Ca^2+^ uptake (RAM) in the liver.

**Fig. 7:**
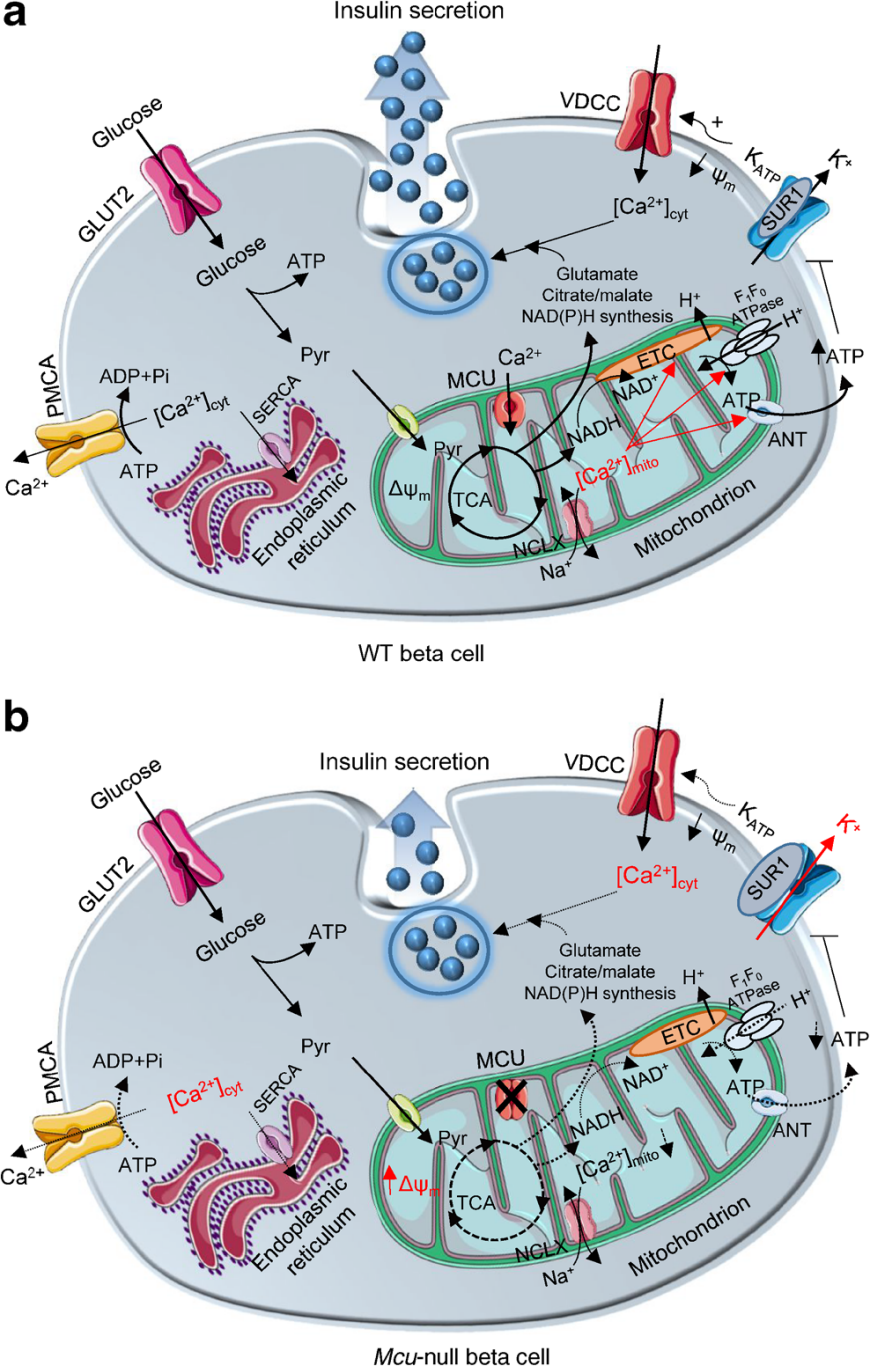
Putative involvement of ΜCU in coordinating the response of beta cells to nutrient supply, and impact of *Mcu* deletion on GSIS. (**a**) In WT animals, glucose is taken up by beta cells and catabolised glycolytically. The formed pyruvate (Pyr) is metabolised by mitochondria through the citrate (TCA) cycle, leading to an increased mitochondrial proton motive force (hyperpolarised Δψ_m_) and accelerated ATP synthesis. By entering mitochondria via the MCU, Ca^2+^ potentiates oxidative metabolism to counter-balance ATP consumption. Ca^2+^ exits mitochondria via NCLX. Consequently, the cytoplasmic ATP:ADP ratio rises, which causes further closure of K_ATP_ channels, depolarisation of plasma membrane potential (ψm), opening of VDCCs and influx of Ca^2+^. Elevated [Ca^2+^]_cyt_ triggers a number of ATP-dependent processes including insulin secretion and Ca^2+^ removal into the endoplasmic reticulum (via the sarco/endoplasmic reticulum Ca^2+^ ATPase [SERCA]) and extracellular medium (plasma membrane Ca^2+^ ATPase [PMCA]), powered by ATP hydrolysis to ADP and inorganic phosphate (Pi). Mitochondrial metabolism is also activated by amino acids, such as glutamate and citrate/malate, which appear to be necessary for appropriate generation of regulatory ‘amplifying’ signals for insulin secretion. (**b**) Following *Mcu* deletion, [Ca^2+^]_mito_ is reduced, leading to a more highly polarised Δψ_m_, weaker oxidative or amino acid metabolism and decreased ATP synthesis, perhaps due to a decrease in mitochondrial F_1_F_0_ATPase and/or adenine nucleotide transferase (ANT) activity. This is expected to result in less closure of K_ATP_ channels, further potentiated by increased expression of the sulfonylurea receptor-1 (SUR1) subunit, weaker ψm depolarisation and Ca^2+^ influx. Importantly, lowered ATP supply to the cytosol is expected to restrict Ca^2+^ pumping across the plasma membrane, as well as into the endoplasmic reticulum. Despite reporting elevated [Ca^2+^]_cyt_ in β*Mcu*-KO mice, insulin secretion in vitro was impaired, possibly due to lower Ca^2+^-dependent intra-mitochondrial generation of putative coupling molecules, such as glutamate and citrate/malate. ETC, electron transport chain. Red font and arrows represent enhanced pathways; dashed arrows represent impaired pathways. This figure was produced using several illustrations from Servier Medical Art, http://smart.servier.com/

Surprisingly, quantification of [Ca^2+^]_cyt_ in intact islets demonstrated larger increases in response to high glucose in KO mouse islets (Fig. 7a,b), perhaps reflecting an impact on Ca^2+^ oscillation frequency, beta cell–beta cell communication and three-dimensional electrical communication through gap junctions [36]. The sharp decrease in GSIS in vitro in the face of higher cytosolic [Ca^2+^] (Fig. 7b) is, again, paradoxical and argues that lowered ATP:ADP, alongside impairments in amplifying processes for insulin secretion [37], such as the Ca^2+^-dependent intra-mitochondrial generation of putative coupling molecules such as glutamate [38] or others [3], exert a dominant inhibitory effect in KO mice.

Importantly, our observations support the view that Ca^2+^ accumulation by mitochondria stimulates ATP consumption, consistent with a reduction in glucose-stimulated ATP:ADP ratio in the KO mouse [7]. Of note, beta cells from β*Mcu*-KO mice tended to be more depolarised than the cells from WT animals at low glucose concentrations, suggesting the entry of positively charged ions (Ca^2+^, Na^+^) within the mitochondrial matrix to allow minimal TCA cycle activity, H^+^ pumping and energy production. Interestingly, during perifusion with high glucose, the Δψ_m_ of dissociated KO mouse-derived beta cells was more polarised than cells from WT animals, suggesting an impaired entrance of the positively charged ions that are necessary for oxidative phosphorylation and ATP production. An increase in Δψ_m_ in the face of lowered cytosolic ATP:ADP is consistent with a decrease in F_1_F_0_ATPase activity [39] (Fig. 7b), an enzyme previously reported to be Ca^2+^ regulated in other tissues [9]. Reduced mitochondrial Ca^2+^ extrusion via flux through the mitochondrial Na^+^/Ca^2+^ exchanger (NCLX) (electrogenic; 3Na^+^: 1Ca^2+^) may also contribute to the increase in Δψ_m_ [40]. Additionally, lowered cytosolic ATP is expected to restrict Ca^2+^ pumping across the plasma membrane (via Ca^2+^ATPase), as well as into the endoplasmic reticulum (via the sarco/endoplasmic reticulum Ca^2+^-ATPase [SERCA]; Fig. 7b). Of note, despite attenuated ATP increases and greater accumulation of cytosolic Ca^2+^, we observed only a lower glucose-stimulated plasma membrane depolarisation and no significant difference in VDCC activity between WT- and KO-derived beta cells. Quantification of K_ATP_ channel subunit expression revealed elevated *Abcc8* mRNA levels in β*Mcu*-KO mice, which, alongside attenuation of the increase in cytosolic ATP:ADP, is expected to lower membrane excitability (Fig. 7b) and Ca^2+^ entry [41].

In addition to the above functional alterations, β*Mcu*-KO mice displayed decreased beta cell mass. This may reflect either impaired proliferation or generation from progenitor cells in the absence of functional *Mcu*, or altered cell death [20]. Of note, Zhao et al. [42] have recently reported that downregulation of MCU enhances autophagic death in neurons due to the activation of AMP-activated protein kinase (AMPK), a known regulator of beta cell mass [43]. Another reason could be an increased production of superoxide anions due to impaired electron transport chain activity (Δψ_m_ and ATP production) affecting beta cell survival, growth and function [44].

Extending to the in vivo setting, the current and earlier [10, 15] in vitro data demonstrating roles for MCU in the control of glucose-induced insulin secretion, we show that insulin secretion is impaired in β*Mcu*-KO vs control mice 5 min post i.p. injection of glucose. Surprisingly, however, insulin secretion post-oral gavage was elevated in KO mice at 5 min, perhaps suggesting that gut-derived factors, such as GIP and the glucagon-like peptide-1 (GLP-1), are partially responsible for the enhanced GSIS from beta cells observed in KO mice [45]. This possibility was explored in vitro by testing the effect of GIP and exendin-4 (a GLP-1 receptor agonist), which are known for promoting insulin secretion [46, 47]. Islets from β*Mcu*-KO mice displayed an impaired sensitivity to incretin hormones, suggesting that GLP-1 signalling is compromised in vitro. Since stimulation of the GLP-1 receptor increases glycolysis and ATP production in beta cells and other tissues through transcriptional activation and expression of glycolytic genes [48], it is reasonable to expect a lowered peptide-induced GSIS in the KO mice in vitro due to an impaired mitochondrial respiratory function. In contrast, following OGTT, insulin secretion was greater in KO animals, with the reasons for this paradoxical result remaining unclear.

Interestingly, earlier studies of inactivating *Mcu* globally in the mouse, or in selected tissues, have consistently reported relatively minor phenotype changes vs controls [49]. Thus, global *Mcu*-null mice display relatively unimpaired cardiac and skeletal muscle function and respiration [12] despite a near-complete ablation of Ca^2+^ accumulation by mitochondria in the cells of these tissues. The present results are in line with these earlier findings. It is, for example, unknown why insulin secretion is acutely impaired 5 min post i.p. glucose administration in KO mice. One intriguing possibility, which may be of particular relevance to the nutrient-responsive beta cell, is that the mitochondrial NCLX operates in the reverse mode at low Δψ_m_, thus allowing Ca^2+^ influx [50], as a compensatory mechanism for the loss of MCU.

The findings here also provide evidence of a role for additional, mitochondria-derived metabolic signals, the generation of which depends on mitochondrial Ca^2+^ uptake, and which serve to potentiate the actions of increased cytosolic Ca^2+^. Such molecules have been proposed to underlie the ‘amplification’ (K_ATP_-channel independent) component of GSIS [37] but still remain elusive. Recent studies have focused on mitochondrial pathways of glucose metabolism, and the generation of second messengers, other than ATP, that such pathways might generate. In particular, a role for anaplerosis is implied by the co-expression of pyruvate carboxylase alongside pyruvate dehydrogenase (PDH) (reviewed previously [3]), and isocitrate export linked to NADPH production is involved in the control of insulin release [51]. Impairments in this and other pathways might, therefore, restrict normal insulin secretion in β*Mcu*-KO mice (Fig. 7b).

### Conclusion

To the best of our knowledge, this study provides only the second description of conditional tissue-restricted *Mcu*-KO mice and reveals a critical role for MCU-mediated mitochondrial Ca^2+^ influx in the pancreatic beta cell in vitro and in vivo. The mechanisms that compensate for defective insulin secretion in vivo in β*Mcu*-KO mice, ensuring near-normal or improved glucose homeostasis, will need further exploration.

Our findings suggest that changes in MCU expression or activity may contribute to defective insulin secretion in some forms of diabetes. An alteration in the ratio of the active (MCU) form of the channel vs MCUb (encoded by a distinct gene, *Mcub*, formerly termed *Ccdc109b*), a dominant-negative form of the carrier [24], might also play a part in the disease process in some settings.

## Electronic supplementary material

ESM 1 (PDF 481 kb)

ESM video 1 Fluorescence imaging of cytosolic Ca2+ oscillations using Cal-520 in WT (left) and βMcu-KO (right) whole islets in response to 3 mmol/l or 17 mmol/l glucose, 17 mmol/l glucose with 0.1 mmol/l diaz or 20 mmol/l KCl with 0.1 mmol/l diaz. Scale bar, 50 μm.(AVI 20014 kb)

ESM video 2 Fluorescence imaging of mitochondrial Ca2+ oscillations using R-GECO in WT (left) and βMcu-KO (right) whole islets in response to 3 mmol/l or 17 mmol/l glucose, 17 mmol/l glucose with 0.1 mmol/l diaz or 20 mmol/l KCl with 0.1 mmol/l diaz. Scale bar, 50 μm.(AVI 27523 kb)

## Data Availability

All data generated or analysed during this study are included in the published article (and its supplementary information files). No applicable resources were generated or analysed during the current study.

## Abbreviations

[Ca^2+^]_cyt_: Cytoplasmic Ca^2+^ concentration
[Ca^2+^]_mito_: Mitochondrial free Ca^2+^ concentration
FCCP: Carbonyl cyanide-4-phenylhydrazone
GIP: Glucose-dependent insulinotropic peptide
GLP-1: Glucagon-like peptide-1
GSIS: Glucose-stimulated insulin secretion
HFHS: High-fat high-sucrose diet
K_ATP_: ATP-sensitive K^+^ (channel)
KO: Knockout
Δψ_m_: Mitochondrial membrane potential
MCU: Mitochondrial Ca^2+^ uniporter
β*Mcu*-KO: Beta cell-specific *Mcu*-null (animal)
MICU: Mitochondrial calcium uptake protein
MOI: Multiplicity of infection
NA: Numerical aperture
NCLX: Na^+^/Ca^2+^ exchanger
OPT: Optical projection tomography
qRT-PCR: Quantitative RT-PCR
RAM: Rapid mode of mitochondrial Ca^2+^ uptake
TCA: Tricarboxylic acid
TMRE: Tetramethylrhodamine ethyl ester
VDCC: Voltage-dependent Ca^2+^ channel
WT: Wild-type

## References

[1] International Diabetes Federation. Type 2 diabetes. Available from www.idf.org/aboutdiabetes/type-2-diabetes.html. Accessed 31 January 2020

[2] Kahn SE, Zraika S, Utzschneider KM, Hull RL (2009). The beta cell lesion in type 2 diabetes: there has to be a primary functional abnormality. Diabetologia.

[3] Rutter GA, Pullen TJ, Hodson DJ, Martinez-Sanchez A (2015). Pancreatic beta-cell identity, glucose sensing and the control of insulin secretion. The Biochemical journal.

[4] Rutter GA, Theler JM, Murgia M, Wollheim CB, Pozzan T, Rizzuto R (1993). Stimulated Ca2+ influx raises mitochondrial free Ca2+ to supramicromolar levels in a pancreatic beta-cell line. Possible role in glucose and agonist-induced insulin secretion. J Biol Chem.

[5] Kennedy ED, Rizzuto R, Theler JM, Pralong WF, Bastianutto C, Pozzan T, Wollheim CB (1996). Glucose-stimulated insulin secretion correlates with changes in mitochondrial and cytosolic Ca2+ in aequorin-expressing INS-1 cells. J Clin Invest.

[6] Denton RM, McCormack JG (1980). On the role of the calcium transport cycle in heart and other mammalian mitochondria. FEBS Lett.

[7] Drews G, Bauer C, Edalat A, Dufer M, Krippeit-Drews P (2015) Evidence against a Ca^2+^-induced potentiation of dehydrogenase activity in pancreatic beta-cells. Pflugers Archiv 467(11):2389–2397. 10.1007/s00424-015-1707-3 10.1007/s00424-015-1707-3 25893711

[8] Wiederkehr A, Wollheim CB (2012). Mitochondrial signals drive insulin secretion in the pancreatic beta-cell. Mol Cell Endocrinol.

[9] Anello M, Lupi R, Spampinato D, Piro S, Masini M, Boggi U, del Prato S, Rabuazzo AM, Purrello F, Marchetti P (2005). Functional and morphological alterations of mitochondria in pancreatic beta cells from type 2 diabetic patients. Diabetologia.

[10] Tarasov AI, Semplici F, Ravier MA, Bellomo EA, Pullen TJ, Gilon P, Sekler I, Rizzuto R, Rutter GA (2012). The mitochondrial Ca2+ uniporter MCU is essential for glucose-induced ATP increases in pancreatic beta-cells. PLoS One.

[11] De Stefani D, Patron M, Rizzuto R (2015). Structure and function of the mitochondrial calcium uniporter complex. Biochim Biophys Acta.

[12] Pan X, Liu J, Nguyen T et al (2013) The physiological role of mitochondrial calcium revealed by mice lacking the mitochondrial calcium uniporter. Nat Cell Biol 15(12):1464–1472. 10.1038/ncb2868 10.1038/ncb2868 PMC3852190 24212091

[13] Hou T, Zhang X, Xu J et al (2013) Synergistic triggering of superoxide flashes by mitochondrial Ca2+ uniport and basal reactive oxygen species elevation. J Biol Chem 288(7):4602–4612. 10.1074/jbc.M112.398297 10.1074/jbc.M112.398297 PMC3576066 23283965

[14] Tarasov AI, Semplici F, Li D et al (2013) Frequency-dependent mitochondrial Ca^2+^ accumulation regulates ATP synthesis in pancreatic β cells. Pflugers Archiv 465(4):543–554. 10.1007/s00424-012-1177-9 10.1007/s00424-012-1177-9 PMC3631125 23149488

[15] Alam MR, Groschner LN, Parichatikanond W (2012). Mitochondrial Ca2+ uptake 1 (MICU1) and mitochondrial Ca2+ uniporter (MCU) contribute to metabolism-secretion coupling in clonal pancreatic β-cells. J Biol Chem.

[16] Hodson DJ, Mitchell RK, Bellomo EA et al (2013) Lipotoxicity disrupts incretin-regulated human beta cell connectivity. J Clin Invest 123(10):4182–4194. 10.1172/jci68459 10.1172/JCI68459 PMC4382273 24018562

[17] Luo J, Deng Z-L, Luo X et al (2007) A protocol for rapid generation of recombinant adenoviruses using the AdEasy system. Nat Protoc 2(5):1236–1247. 10.1038/nprot.2007.135 10.1038/nprot.2007.135 17546019

[18] Wu J, Prole DL, Shen Y et al (2014) Red fluorescent genetically encoded Ca2+ indicators for use in mitochondria and endoplasmic reticulum. The Biochemical journal 464(1):13–22. 10.1042/bj20140931 10.1042/BJ20140931 PMC4214425 25164254

[19] Zhu L, Almaca J, Dadi PK (2017). β-Arrestin-2 is an essential regulator of pancreatic β-cell function under physiological and pathophysiological conditions. Nat Commun.

[20] Sun G, Tarasov AI, McGinty J (2010). Ablation of AMP-activated protein kinase alpha1 and alpha2 from mouse pancreatic beta cells and *RIP2.Cre* neurons suppresses insulin release in vivo. Diabetologia.

[21] Thorens B, Tarussio D, Maestro MA, Rovira M, Heikkila E, Ferrer J (2015). *Ins1*^Cre^ knock-in mice for beta cell-specific gene recombination. Diabetologia.

[22] Elayat AA, el-Naggar MM, Tahir M (1995). An immunocytochemical and morphometric study of the rat pancreatic islets. J Anat.

[23] Benner C, van der Meulen T, Caceres E, Tigyi K, Donaldson CJ, Huising MO (2014). The transcriptional landscape of mouse beta cells compared to human beta cells reveals notable species differences in long non-coding RNA and protein-coding gene expression. BMC Genomics.

[24] Mammucari C, Raffaello A, Vecellio Reane D, Rizzuto R (2016). Molecular structure and pathophysiological roles of the mitochondrial calcium uniporter. Biochim Biophys Acta.

[25] Ashcroft FM, Rorsman P (2013). K_ATP_ channels and islet hormone secretion: new insights and controversies. Nat Rev Endocrinol.

[26] Tarasov AI, Griffiths EJ, Rutter GA (2012). Regulation of ATP production by mitochondrial Ca^2+^. Cell Calcium.

[27] Wiederkehr A, Szanda G, Akhmedov D (2011). Mitochondrial matrix calcium is an activating signal for hormone secretion. Cell Metab.

[28] Civelek VN, Deeney JT, Shalosky NJ (1996). Regulation of pancreatic β-cell mitochondrial metabolism: influence of Ca^2+^, substrate and ADP. The Biochemical journal.

[29] Mulder H, Ling C (2009). Mitochondrial dysfunction in pancreatic β-cells in type 2 diabetes. Mol Cell Endocrinol.

[30] Ma ZA, Zhao Z, Turk J (2012). Mitochondrial dysfunction and beta-cell failure in type 2 diabetes mellitus. Exp Diabetes Res.

[31] Denton RM, Pullen TJ, Armstrong CT, Heesom KJ, Rutter GA (2016) Calcium-insensitive splice variants of mammalian E1 subunit of 2-oxoglutarate dehydrogenase complex with tissue-specific patterns of expression. BIochem J 473(9):1165–1178. 10.1042/BCJ20160135 10.1042/BCJ20160135 PMC6101200 26936970

[32] Kamer KJ, Mootha VK (2014). MICU1 and MICU2 play nonredundant roles in the regulation of the mitochondrial calcium uniporter. EMBO Rep.

[33] Wang Y, Nguyen NX, She J, Zeng W, Yang Y, Bai XC, Jiang Y (2019). Structural mechanism of EMRE-dependent gating of the human mitochondrial calcium uniporter. Cell.

[34] Bondarenko AI, Jean-Quartier C, Malli R, Graier WF (2013) Characterization of distinct single-channel properties of Ca^2+^ inward currents in mitochondria. Pflugers Archiv 465(7):997–1010. 10.1007/s00424-013-1224-1 10.1007/s00424-013-1224-1 PMC3696464 23397170

[35] Jakob R, Beutner G, Sharma VK etal (2014) Molecular and functional identification of a mitochondrial ryanodine receptor in neurons. Neurosci Lett 575:7–12. 10.1016/j.neulet.2014.05.026 10.1016/j.neulet.2014.05.026 PMC4122666 24861510

[36] Rutter GA, Hodson DJ (2015). Beta cell connectivity in pancreatic islets: a type 2 diabetes target?. Cell Mol Life Sci.

[37] Henquin JC (2000). Triggering and amplifying pathways of regulation of insulin secretion by glucose. Diabetes.

[38] Maechler P, Wollheim CB (1999). Mitochondrial glutamate acts as a messenger in glucose-induced insulin exocytosis. Nature.

[39] Balaban RS (2009). The role of Ca^2+^ signaling in the coordination of mitochondrial ATP production with cardiac work. Biochim Biophys Acta.

[40] Palty R, Silverman WF, Hershfinkel M et al (2010) NCLX is an essential component of mitochondrial Na+/Ca2+ exchange. Proc Natl Acad Sci U S A 107(1):436–441. 10.1073/pnas.0908099107 10.1073/pnas.0908099107 PMC2806722 20018762

[41] Fridlyand LE, Jacobson DA, Philipson LH (2013). Ion channels and regulation of insulin secretion in human beta-cells: a computational systems analysis. Islets.

[42] Zhao M, Chen J, Mao K etal (2019) Mitochondrial calcium dysfunction contributes to autophagic cell death induced by MPP^+^ via AMPK pathway. Biochem Biophys Res Commun 509(2):390–394. 10.1016/j.bbrc.2018.12.148 10.1016/j.bbrc.2018.12.148 30594390

[43] Kefas BA, Heimberg H, Vaulont S et al (2003) AICA-riboside induces apoptosis of pancreatic beta cells through stimulation of AMP-activated protein kinase. Diabetologia 46(2):250–254. 10.1007/s00125-002-1030-3 10.1007/s00125-002-1030-3 12627324

[44] Drews G, Krippeit-Drews P, Düfer M (2010) Oxidative stress and beta-cell dysfunction. Pflugers Arch - Eur J Physiol 460(4):703–718. 10.1007/s00424-010-0862-9 10.1007/s00424-010-0862-9 20652307

[45] Salehi M, Aulinger B, D’Alessio DA (2012). Effect of glycemia on plasma incretins and the incretin effect during oral glucose tolerance test. Diabetes.

[46] Drucker DJ (2006). The biology of incretin hormones. Cell Metab.

[47] Hansotia T, Drucker DJ (2005). GIP and GLP-1 as incretin hormones: lessons from single and double incretin receptor knockout mice. Regul Pept.

[48] Carlessi R, Chen Y, Rowlands J et al (2017) GLP-1 receptor signalling promotes β-cell glucose metabolism via mTOR-dependent HIF-1α activation. Sci Rep 7(1):2661. 10.1038/s41598-017-02838-2 10.1038/s41598-017-02838-2 PMC5454020 28572610

[49] Harrington JL, Murphy E (2015). The mitochondrial calcium uniporter: mice can live and die without it. J Mol Cell Cardiol.

[50] Samanta K, Mirams GR, Parekh AB (2018). Sequential forward and reverse transport of the Na^+^ Ca^2+^ exchanger generates Ca^2+^ oscillations within mitochondria. Nat Commun.

[51] Ferdaoussi M, Dai X, Jensen MV et al (2015) Isocitrate-to-SENP1 signaling amplifies insulin secretion and rescues dysfunctional beta cells. J Clin Invest 125(10):3847–3860. 10.1172/jci82498 10.1172/JCI82498 PMC4607115 26389676

