# Supplementary material for: The pore-forming subunit MCU of the mitochondrial Ca2+ uniporter is required for normal glucose-stimulated insulin secretion in vitro and in vivo in mice
Source: Diabetologia. 2020 Apr 29;63(7):1368–81. doi: 10.1007/s00125-020-05148-x (PMC7286857; doi:10.1007/s00125-020-05148-x)
Supplement: Supplementary file 1 — (PDF 481 kb) [file 125_2020_5148_MOESM1_ESM.pdf]

ESM Methods

SDS-PAGE and western blotting

After isolation, ~100 islets from WT or *βMcu*-KO mice were collected and lysed in ice-cold buffer (150 mmol/l NaCl, 10 mmol/l Tris HCl pH 7.2, 0.1% SDS, 1% sodium deoxycholate, 5 mmol/l EDTA, 1% Triton X-100) containing protease inhibitor cocktail (Roche) and phosphatase inhibitors (Sigma-Aldrich). 20 μg of total proteins were loaded, according to bicinchoninic acid (BCA) assay (Thermo Fisher) quantification. Lysates were denatured and resolved by 12% SDS-PAGE, and transferred to poly-vinylidene fluoride (PVDF) membranes (GE Healthcare) before immunoblotting. Blots were blocked 1 h at room temperature (RT) with 5% non-fat dry milk (Sigma-Aldrich) in TBS-tween (10 mmol/l Tris, 150 mmol/l NaCl, 0.1% Tween 20) solution and incubated at 4 °C with the primary antibody. Secondary antibodies were incubated 1 h at RT. Antibodies used in Western (immuno-) blot analysis were the following: rabbit anti-MCU (Sigma-Aldrich, HPA016480, 1:1000), goat anti-rabbit GAPDH (Cell signalling, 1:10 000), goat anti-rabbit HRP (Abcam, 1:5000). Validation of MCU antibody was determined by MCU deletion in the islets (Fig.1b).

mRNA extraction and qRT-PCR

RNA was extracted from 8-10 week male mouse islets, liver, heart and adipose tissues using Trizol (Invitrogen) and reverse transcribed using a high capacity reverse transcription kit (Invitrogen) [1][2]. Gene expression was determined by qRT-PCR using SYBR Green (Invitrogen) and normalised to *β-actin*. The sequences of primers used for genotyping and qRT-PCR for *Mcu, Micu1-3, Smdt1, Kcnj11* and *Abcc8* are provided under Supplemental Tables 1 and 2.

Intraperitoneal glucose, insulin tolerance tests and measurement of insulin secretion in vivo

To investigate glucose tolerance, male or female mice (ages 8-24 weeks as indicated) were fasted overnight for 16 h before i.p injection of glucose solution (20% w/v, 1g/kg body weight). Glucose was measured in tail vein blood at 0, 5, 15, 30, 60, 90 and 120 min. using an ACCU-CHECK Aviva glucometer (Roche) [3].

To ascertain insulin tolerance, 8-10 week male mice were fasted for 5 h before human insulin (0.75 units/kg body weight, Sigma Aldrich) was injected intraperitoneally. Blood glucose was measured in tail vein blood at 0, 15, 30 and 60 min. [3].

For in vivo insulin secretion experiments, 8-10 week males were fasted overnight for 16 h and glucose (20% w/v, 3 g/kg body weight) was either given intraperitoneally or oral gavage. Plasma was separated by centrifugation and insulin was measured using an ultra-sensitive mouse insulin enzyme-linked immunosorbent assay (ELISA) kit (CrystalChem).

Electrophysiology

Electrophysiological recordings were performed on single beta cells isolated from 8-10 week males in perforated patch-clamp configuration using an EPC9 patch-clamp amplifier controlled by Pulse acquisition software (Heka Elektronik, Pfalz, Germany). Beta cells were identified morphologically and by depolarisation of the membrane potential in response to 17 mmol/l glucose. Beta cells were constantly perfused at 32 °C with normal saline solution (135 mmol/l NaCl, 5 mmol/l KCl, 1 mmol/l $MgCl_2$, 1 mmol/l $CaCl_2$, 10 mmol/l HEPES, pH 7.4). Recording electrodes had resistances of 8-10 MΩ and were filled with a solution comprised of 140 mmol/l KCl, 5 mmol/l $MgCl_2$, 3.8 mmol/l $CaCl_2$, 10 mmol/l HEPES, 10 mmol/l EGTA (pH 7.2) and 20–25 μg/ml amphotericin B (Sigma-Aldrich).

ESM tables

ESM Table 1.

Mcu (Mcua)	F: 5’CTGCTTCTGTGTACATTCAAGGATG
	R: 5’CTCGGTTCTAGATACTGGCATTCAC

Sequences of primers used for genotyping of *Mcua* flox. (F: forward, R: reverse).

ESM Table 2.

qRT-PCR	*Mcu*	F-GAGCAGCATCAGCTTAACAAAGAG
		R-TCTGCTAATTTCAATTCGTACCTTCTC
	β-actin	F-CGAGTCGCGTCCACCC
		R-CATCCATGGCGAACTGGTG
	Abcc8	F-GCCTACGCATCTCAGAAACCA
		R-CCATCTTGTACCTTTGCTTATTGAAG
	Kcnj11	F-CACGGCGGGATAAGTCTACCT
		R-AATCATTTGCCCCCTTCTTGT
	Micu1	F-CGGCATTTGCGTCTCTATGG
		R-CTTCGTTTGGTCTGTGTGGG
	Micu2	F-CGGTGTCGGCGCAGAAAAAT
		R-TGAGAAGAGGAAGTCTCGGG
	Micu3	F-ATTAAGTCAGATGCTCTCAG
		R-TCTGTGTAAGAAATCACACC
	Smdt1	F-GAACTTCGCTGCTCTGCTTG
		R-CAAACCATGAGGAGGTGAGG

List of primers used for qRT-PCR amplification (F: forward, R: reverse).

ESM figures

ESM Fig. 1

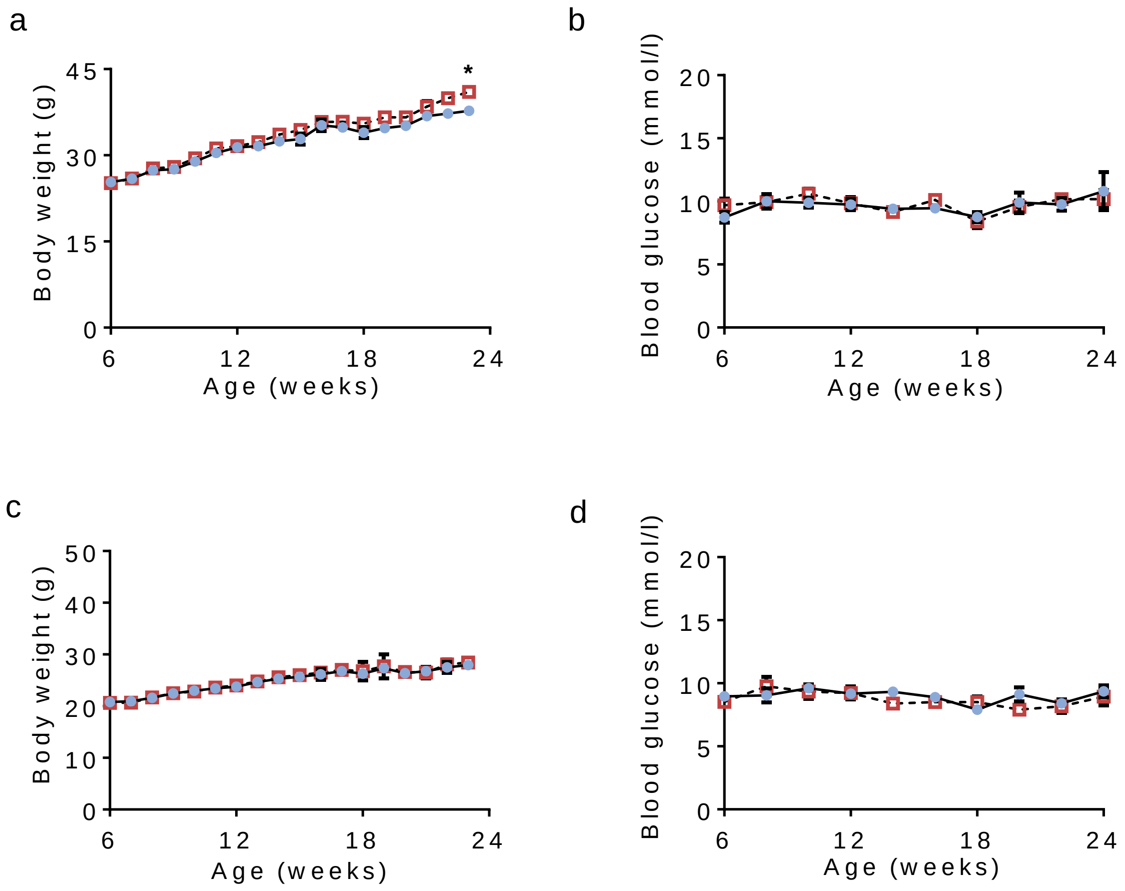

Male but not female β*Mcu*-KO mice display slightly increased weight gain on standard chow diet, compared to littermate controls. (a) Body weight and (b) random fed glycaemic profile of male and (c, d) female β*Mcu*-KO and littermate control mice (WT). (a,b) (*n*=11-14 mice per genotype of two or four independent experiments; two-way ANOVA and Bonferroni correction for multiple tests) and (c,d) (*n*=10-12 mice per genotype of two or four independent experiments; two-way ANOVA and Bonferroni correction for multiple tests) accordingly. Blue, WT mice; red, β*Mcu*-KO mice. Values represent as mean±SEM. Significance was determined between WT and KO mice at the time points indicated *$p<0.05$.

ESM Fig. 2

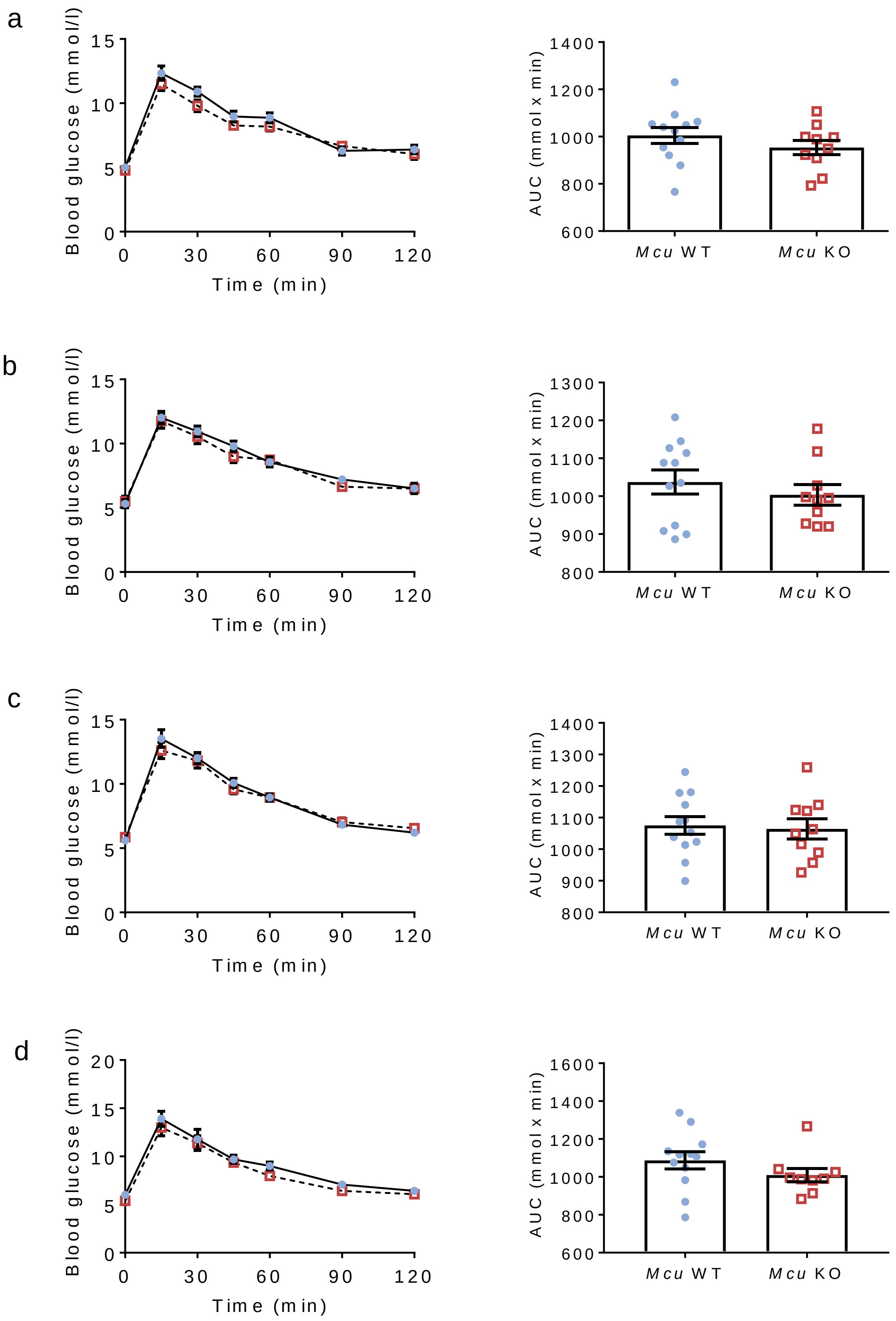
a
b
c
d
Blood glucose (mmol/l)
Time (min)
0
30
60
90
120
AUC (mmol x min)
Mcu WT
Mcu KO

Female β*Mcu*-KO mice display normal glucose tolerance. (a) Glucose tolerance was measured in female β*Mcu*-KO and littermate control (WT) mice by i.p injection of glucose (1g/kg body weight) at 8, (b) 12, (c) 16 and (d) 24 weeks of age (*n*=10-12 mice per genotype of two or four independent experiments; two-way ANOVA and Bonferroni correction for multiple tests). The AUC is shown to the right of each graph (*n*=10-12 mice per genotype of two or four independent experiments; unpaired Student's t-test). All mice were maintained on a standard chow diet. Values represent mean ± SEM.

ESM Fig. 3

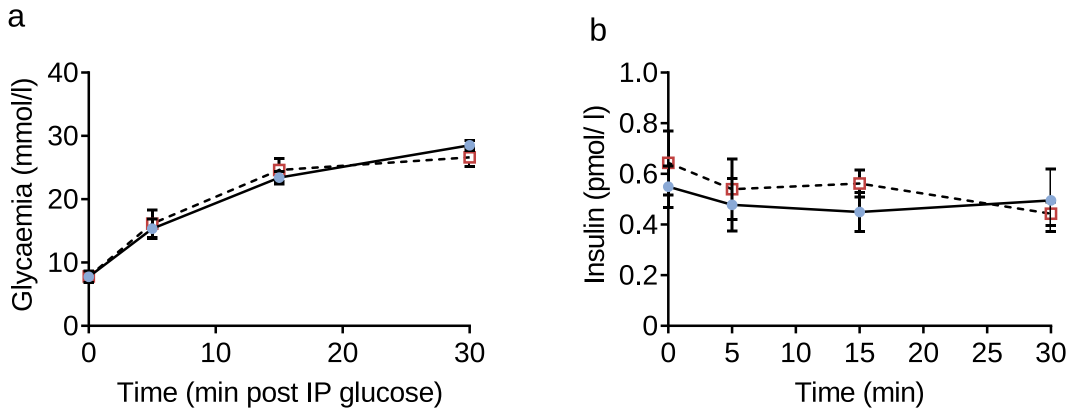

Male β*Mcu*-KO mice display normal glucose tolerance and insulin secretion following a HFHS diet. (a) Glycaemia and (b) glucose (3 g/kg body weight)-induced insulin secretion were assessed in β*Mcu*-KO and WT mice (8 week old mice; *n*=5-7 mice per genotype of two independent experiments; two-way ANOVA and Bonferroni correction for multiple tests). Values represent mean ± SEM.

ESM video legends

ESM video 1

Fluorescence imaging of cytosolic Ca^{2+} oscillations using Cal-520 in WT (left) and β*Mcu*-KO (right) whole islets in response to 3 mmol/l or 17 mmol/l glucose, 17 mmol/l glucose with 0.1 mmol/l diaz or 20 mmol/l KCl with 0.1 mmol/l diaz. Scale bar, 50 μm.

ESM video 2

Fluorescence imaging of mitochondrial Ca^{2+} oscillations using R-GECO in WT (left) and β*Mcu*-KO (right) whole islets in response to 3 mmol/l or 17 mmol/l glucose, 17 mmol/l glucose with 0.1 mmol/l diaz or 20 mmol/l KCl with 0.1 mmol/l diaz. Scale bar, 50 μm.

References

[1] Ravier MA, Rutter GA (2010) Isolation and culture of mouse pancreatic islets for ex vivo imaging studies with trappable or recombinant fluorescent probes. Methods in molecular biology (Clifton, NJ) 633: 171-184. 10.1007/978-1-59745-019-5_12

[2] Martinez-Sanchez A, Nguyen-Tu MS, Rutter GA (2015) DICER Inactivation Identifies Pancreatic beta-Cell "Disallowed" Genes Targeted by MicroRNAs. Molecular endocrinology (Baltimore, Md) 29(7): 1067-1079. 10.1210/me.2015-1059

[3] Mehta ZB, Fine N, Pullen TJ, et al. (2016) Changes in the expression of the type 2 diabetes-associated gene VPS13C in the beta-cell are associated with glucose intolerance in humans and mice. American journal of physiology Endocrinology and metabolism 311(2): E488-507. 10.1152/ajpendo.00074.2016
